# Evaluation of Hormonal Profile and Ovarian Morphology among Adolescent Girls with Menstrual Irregularities in a Tertiary Care Centre at Central India

**DOI:** 10.1155/2022/3047526

**Published:** 2022-07-15

**Authors:** Shweta Patel, K. Pushpalatha, Bharti Singh, Ragini Shrisvastava, Gyanendra Singh, Deepti Dabar

**Affiliations:** ^1^Department of Obstetrics and Gynecology, All India Institute of Medical Sciences, Bhopal, M.P., India; ^2^Department of Physiology, All India Institute of Medical Sciences, Bhopal, M.P., India; ^3^Department of Community and Family Medicine, All India Institute of Medical Sciences, Bhopal, M.P., India

## Abstract

Menstrual disturbances are common among adolescents with a prevalence rate of 11.3–26.7%. The most frequent menstrual irregularities are oligomenorrhea, menorrhagia, polymenorrhoea, and hypomenorrhea. PCOS (polycystic ovarian syndrome) is now recognized as the most prevalent endocrine disorder among the women of reproductive age. The current study was planned to evaluate socio-demographic factors, endocrine profiles, and ovarian morphology among adolescent girls with menstrual irregularities and compare these parameters in different phenotypes of adolescent PCOS cases. It is a hospital-based cross-sectional study among 248 adolescent girls (10–19 years) with menstrual irregularities. After obtaining informed consent, history and clinical examination findings were recorded on preform proforma. All girls were assessed on day 2/3 of the menstrual cycle for hormonal profile (serum TSH, FSH, LH, prolactin, and serum testosterone) and ovarian morphology (by transabdominal ultrasonography). All participating girls were divided into three groups (groups 1, 2, and 3) corresponding to phenotypes A, B, & D as per the Rotterdam criteria. In the study, oligomenorrhea was the most common menstrual disorder (70.97%). Biochemical hyperandrogenism and thyroid dysfunction were reported in 14.91% and 8.46% of girls, respectively. Our study noted that phenotype D ,i.e., group 3 (MI + PCOM-HA; 49.43%) was the most common phenotype in the study. In a comparative analysis of different groups, significant differences (*p* < 0.05) in hormonal and metabolic parameters showed highest in group 2, which represents phenotype B of PCOS (hyperandrogenic anovulation). This analysis revealed that adolescent hyperandrogenism (phenotypes A and B) is associated with a more deranged hormonal and metabolic profile than nonandrogenic PCOS (phenotype D). To prevent long-term sequelae, lifestyle changes, early treatment, and close follow-up are recommended in this subset of girls.

## 1. Introduction

Polycystic ovary syndrome is an endocrine condition that is characterized by typical hormonal & metabolic features. With a global preference of around 5%–18% of women in reproductive age and 6%–18% of adolescent girls, it is one of the most common health issues faced by women & girls all across the globe. It is a heterogeneous androgen-excess disorder with a typical presentation of different degrees of reproductive and metabolic dysfunctions; it can also be accompanied by other health-related issues such as insulin resistance and metabolic syndrome [1].

Menstrual abnormalities are more common among younger girls and become less frequent as they grow older, 3–5 years after menarche [2, 3]. Occasional deviations usually have temporary causes, such as psychological or physical stress, while chronic anomalies are much more likely to have pathological causes, the most common being polycystic ovary syndrome [PCOS]. Although this condition is now recognized as the most common endocrine abnormality in women of reproductive age, diagnosis of PCOS during adolescence is both controversial and challenging with adult PCOS criteria, due to the overlap of normal pubertal physiological changes (irregular menstrual cycles, acne, and polycystic ovarian morphology on pelvic ultrasound).

As defined by the World Health Organisation, adolescence is the period between 10 and 19 years of age that includes significant and critical changes in growth, development, and puberty due to the overlap of normal pubertal physiological changes such as irregular menstrual cycles, acne, and polycystic ovarian morphology on pelvic ultrasound with the PCOS diagnostic criteria [4]. The first comprehensive definition of PCOS was given by Stein and Leventhal in the year 1935. With a diverse range of clinical manifestations, unspecified etiology, and complex pathophysiology, the diagnosis of PCOS has always been a topic of scientific debate [5].

However, at present, there are 3 different criteria for the diagnosis of PCOS. All require the exclusion of other disorders such as congenital adrenal hyperplasia and tumours [6].

**Figure 1:**
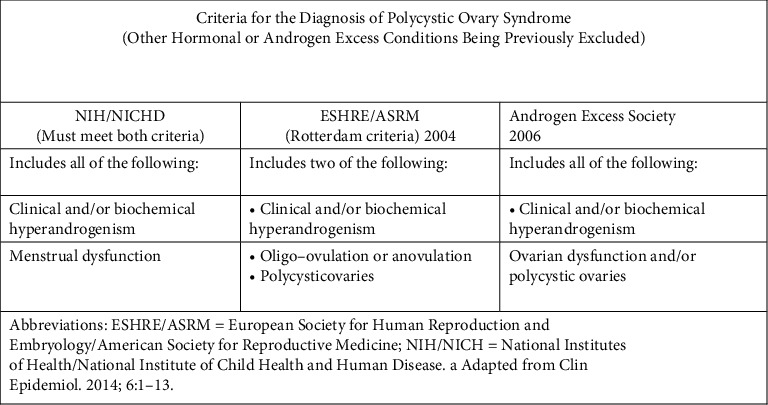
Diagnostic criteria for polycystic ovary syndrome [8].

Guidelines for diagnosis of PCOS in adult women comprise criteria laid down by the National Institute of Health (NIH) in 1990, the Rotterdam criteria was laid down in 2003, and the Androgen Excess and Polycystic Ovary Syndrome Society (AE-PCOS) criteria (Figure 1) [7].

**Figure 2:**
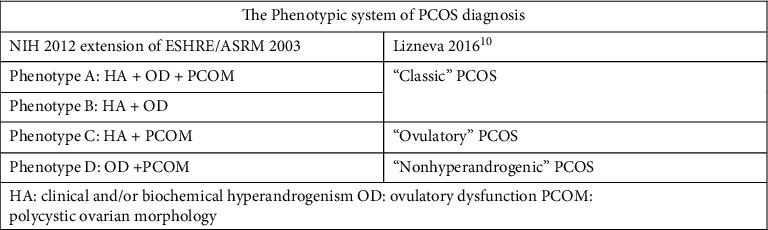
Phenotypes of polycystic ovary syndrome as per the Rotterdam criteria [11].

The diagnosis of PCOS in adolescents should be based on the presence of clinical and/or biochemical hyperandrogenism and irregular menses at least 2 years post menarche; PCOM (polycystic ovarian morphology) on ultrasound is not a diagnostic criterion in adolescents [9]. In 2016, Lizneva et al. further refined the phenotypic approach [10]. They proposed three PCOS phenotypes based on the 2012 modified Rotterdam criteria: “classic” PCOS (phenotypes A/B), “ovulatory PCOS” (phenotype C), and “nonhyperandrogenic PCOS” (phenotype D) (Figure 2). This system more accurately represents PCOS as a true spectrum of findings with variable severity and long-term effect [8].

## 2. Objectives

1. To study socio-demographic and clinical characteristics of adolescent girls with menstrual irregularities
2. To identify various endocrinal profiles in adolescents with menstrual irregularities
3. To assess clinical characteristics of different phenotypic groups of adolescents with PCOS
4. To compare the endocrinal and metabolic parameters in different phenotypes of adolescents with PCOS

## 3. Material & Methods

This present cross-sectional study was undertaken among adolescent girls (10–19 years) with menstrual irregularities visiting the outpatient department of obstetrics & gynaecology department from August 2019 to October 2021 at a tertiary care institute in central India. Adolescent girls (10–19 years) who presented to the outpatient department with a complaint of menstrual irregularities 2 years post menarche were enrolled in the study. Girls with primary amenorrhea, virilization, physical or psychological disease, and chronic illness or pregnancy complications were excluded from the study. The calculated sample size to estimate the prevalence of menstrual irregularity with a 95% confidence interval was 213. The final sample size was 234 with a 10% nonresponse rate. After obtaining written informed consent (herself/parents/guardians) socio-demographic factors, menstrual patterns, relevant past medical, and family history, and clinical examination finding such as acne and hirsutism were recorded on the preformed proforma. Height (meters) and weight (kgs) were measured using standard methods and BMIs were calculated. All girls were assessed on day 2/3 of the menstrual cycle or after withdrawal bleeding for hormonal and metabolic profile (serum TSH, FSH, LH, prolactin, testosterone, fasting blood sugar, and fasting insulin) and ovarian morphology (using transabdominal ultrasonography) as a standard of care.

Enrolled girls after excluding other endocrinopathies were further classified into different phenotypical groups based on polycystic ovaries and clinical (C) and/or biochemical (B) hyperandrogenism. Clinical characteristics along with endocrinal and metabolic profiles were compared in these phenotypes of PCOS.

The phenotypic groups in the study were as follows:

  Group 1: menstrual irregularities (MI) + hyperandrogenemia (HA(C/B)) + polycystic ovarian morphology (PCOM)
  Group 2: menstrual irregularities (MI) + hyperandrogenemia (HA(C/B))—polycystic ovarian morphology (PCOM)
  Group 3: menstrual irregularities (MI) + polycystic ovarian morphology (PCOM)—hyperandrogenemia (HA(C/B))

A transabdominal ultrasound examination was done to measure the ovarian volume during the early follicular phase of the cycle (d1-5) by using a convex array transducer with a bandwidth of 2–5 MHz. The ovarian volume was calculated by using the simplified formula for a prolate ellipsoid i.e., 0.5 × length × width × thickness. Ultrasonographic findings of polycystic ovaries were considered as having 12 or more follicles measuring between 2 and 9 mm and/or an ovarian volume ≥10 cm^3^ [11].

Data analysis was done with SPSS v23 (IBM Corp). Descriptive analysis for the categorical variables was performed by percentage and counts. Numerical and continuous variables were presented as mean. Comparison of categorical data was conducted by using the chi-square test, and one-way analysis of variance (ANOVA) was used for comparative analysis of the mean among different groups = in order to indicate a statistically significant difference *P* < 0.05 was considered.

### 3.1. Operational Definitions

  Socioeconomic status was assessed by using a modified Kuppuswamy scale. Families were classified into the following classes: [12]
  upper: 26–29; upper-middle: 16–25; lower-middle: 11–15; upper-lower: 5–10; lower: 01–04.
  Body mass index (BMI) was calculated by taking a person's weight, in kilograms, divided by their height, in meters square, BMI = weight [kg]/height [m]^2^.
  Underweight—BMI <18.5 kg/m^2^; normal weight—BMI ≥18.5–23 kg/m^2^; overweight—BMI between ≥23–27.5 kg/m^2^; obese—BMI ≥27.5 kg/m^2^ [12].
  Oligomenorrhea is defined as infrequent menstruation that occurs at intervals of >45 days in adolescents [13]
  Polymenorrhagia is defined as frequent episodes of menstruation usually occurring at intervals of <21 days [13]
  Hypomenorrhea is defined as regularly timed but scanty episodes of bleeding [13]
  Menorrhagia: is defined as regularly timed episodes of bleeding that are excessive in amount (>80 ml) and/or duration of flow (>5 days) [13]
  Metriopathei is: defined as episodes of amenorrhea followed by excessive bleeding [13]
  Clinical hyperandrogenism: hirsutism was assessed by Ferriman–Gallwey with the score of ≥8 over 9 body parts [14]. Acne was assessed as numerous papules and pustules, and occasional inflamed nodules on the face, chest, and back [15].
  Biochemical hyperandrogenism was assessed by serum testosterone level of ≥82 ng/dl in the absence of other causes of hyperandrogenism [16]
  Thyroid dysfunction was assessed by a serum level of thyroid outside the range of normal as free T3—2.3–4.2 pg/ml, free T4—0.8–2.0 ng/dl, and TSH—0.7–6.4 *μ*IU/ml
  Hyperprolactinemia was assessed by a serum prolactin level in the blood of >25 ng/ml

## 4. Result

**Figure 3:**
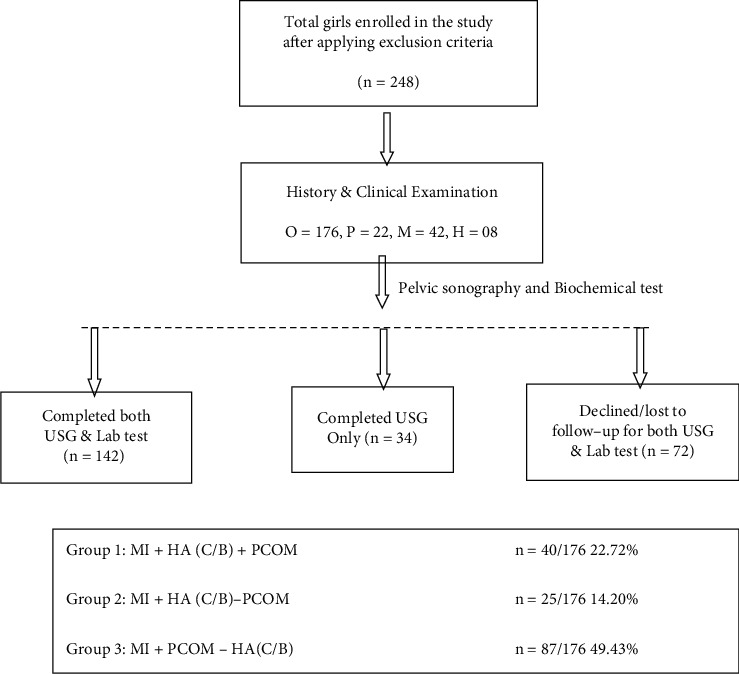
Study flow chart.

In the present study, 248 girls (age group 10–19 years) with menstrual irregularities 2 years post-menarche were enrolled. As illustrated in Table 1, the mean age of the study participants was 17.08 ± 1.91 years with the mean age at menarche being 11.97 ± 1.02 years. In the study, 70.97% of girls had oligomenorrhoea as a major complaint for reporting to the outpatient department. Other menstrual irregularities were polymenorrhoea, menorrhagia/menometrorrhagia, and hypomenorrhea in 8.87%, 16.93%, and 3.22% of girls, respectively. Of all the enrolled girls, 142 had completed both pelvic ultrasonography and biochemical tests after taking relevant clinical history, and 34 had undergone USG only. 72 girls were either not reported or denied for investigations after clinical examination in the study (Figure 3).

According to the Rotterdam criteria laid down for the classification of groups in the enrolled cases, 40 (22.72%) girls were classified in group 1(MI + HA (C/B) + PCOM), 25 (14.20%) in group 2, (MI + HA (C/B)-PCOM) and 87 (49.53%) in group 3 (MI + PCOM—HA(C/B). Group 1 represents phenotype A, group 2 represents phenotype B, and group 3 represents phenotype D of Rotterdam's classification, respectively (Table 2). The most prevalent phenotype in our study was phenotype D, i.e., group 3 (49.43%). The majority of girls in our study represent upper, upper-middle, and lower-middle classes of the socioeconomic status as per the modified Kuppuswami classification. In the study, family history of menstrual disorder was present in 34.67% of girls.

In all adolescent girls with menstrual irregularities, biochemical hyperandrogenism was reported in 14.91% of girls where oligomenorrhoea was the most prevalent menstrual irregularity. Thyroid dysfunction and hyperprolactinemia were reported in 8.46% and 1.20% of girls, respectively (Table 2).

The clinical characteristics of different groups are summarized in Table 3. There was a statistically significant difference in the family history of PCOS and the history of weight gain in the study. PCOS history in first degree relatives was present in 65% and 60% of girls in groups 1 and 2, respectively. Group 2 showed that a majority of girls (56%) reported a history of weight gain in the last year. High BMI (overweight and obese) was more prevalent in group 1 (67.5%), followed by group 2 (60.0%). For hyperandrogenism in group 1, moderate to severe acne is the major symptom (50.0%). High testosterone was noted in 40.0% of girls and hirsutism was reported in 32.5% of girls, whereas in group 2, hirsutism was reported in 48.0% of girls. Most of the girls have bilateral polycystic ovaries on pelvic ultrasound (92.5% in group 1; 77.01% in group 3).

Comparative analysis of endocrinal, metabolic, and hormonal profiles of different groups is shown in Table 4. There were significant differences (*p* < 0.05) noted S. LH, S. testosterone level, BMI, S. fasting glucose, and S. fasting insulin. All parameters were high in group 2, which represents phenotype B of PCOS (hyperandrogenic anovulation). The S. FSH level showed no significant difference in the groups.

## 5. Discussion

It is well established by a plethora of literature that polycystic ovarian syndrome (PCOS) is highly prevalent among women of reproductive age. Considering the importance of PCOS on reproductive health and the associated increased risk leading to other comorbid conditions, the present study was carried out to investigate the clinical profile of adolescent girls with menstrual irregularities in a tertiary care centre in an urban locality.

In the present study, oligomenorrhea (70.96%) was commonly reported as a menstrual irregularity by these girls at our centre. A study by De Leo et al. reported oligomenorrhea in 43% of adolescent girls while Rajiwade et al. reported its prevalence as 61% [17].

As our center is an urban healthcare tertiary referral centre, most girls in our study were from the lower-middle (39.91%]) and upper-middle (32.25%) class of the socioeconomic status. The most common phenotype in our study was phenotype D i.e., group 3(MI + PCOM-HA; 49.43%), followed by phenotype A i.e., group 1 (MI + HA + PCOM; 22.72%), and phenotype B i.e., group 2 (MI + HA-PCOM; 14.20%]) Similar to our study, Pikee et al. found the most common phenotype to be D in a north Indian population [18]. Joshi et al. from Western India reported phenotype D in 52.6% of the study population [19]. The prevalence of these phenotypes differs between published studies across the world. Defer to our results, other studies reported phenotype A as the most prevalent phenotype, and the prevalence varied from 44.09 to 60%. [20–22]. This difference in the prevalence of different phenotypes was probably due to genetic, ethical, cultural, and socio-economical differences across different geographic areas.

The endocrinal profiles of adolescent girls with menstrual irregularities are summarized in Table 2. Thyroid dysfunction was present in 07.38%, biochemical hyperandrogenism in 14.91%, and hyperprolactinemia in 1.20% of girls with menstrual irregularities. In agreement with our result study, Rajiwade et al. reported thyroid dysfunction, hyperandrogenism, and hyperprolactinemia in 13.6%, 09.04%, and 0.94% of girls with menstrual irregularities, respectively [13].

Our study (Table 3) showed a significant association between family history of PCOS and history of weight gain in all phenotypes (group1, group 2, and group 3) of enrolled girls which is supported by the literature as familial clusters are common in PCOS because of complex genetic association [23]. BMI of ≥23 (Obesity and overweight) was reported in 67.5% and 60% of girls presented with clinical or biochemical hyperandrogenemia (groups 1 and 2, respectively). Thathapudi et al. found a higher prevalence of obesity (70%) in phenotype A compared to other phenotypes [24]. It is considered that a vicious cycle between androgen excess and abdominal deposition of visceral fat results in PCOS [25]. In our study, acne was predominant (group 1: 20.0%; group 2: 44.0%) than hirsutism (group 1: 32.5%; group 2: 48.0%) as clinical hyperandrogenism. Biochemical hyperandrogenism was noted in 40.0% and 36.0% of girls in group 1 and 2, respectively. Symptoms related to androgen excess such as hirsutism may be less frequent in adolescents when compared with adults due to the relatively short-term exposure to elevated circulating androgen levels and different peripheral sensitivity of androgen receptors [11, 17]. Although acne is a common presentation as a clinical sign of hyperandrogenism in our study, many other studies [11, 17] suggested that hirsutism and biochemical hyperandrogenemia are more reliable markers in adolescents for defining PCOS, and they should not be used in isolation to define hyperandrogenism in adolescents [26]. De Leo et al. reported that about 50% of women with acne have no clinical or biochemical evidence of hyperandrogenism [17]. Further longitudinal studies are required for future clarification of these results.

Bilateral polycystic ovaries were present in 92.5% of girls in group 1 and 77.01% in group 3. The right bulky polycystic ovary was present in 16.09% and 07.5% of girls in group 3 and group 1, respectively. In contrast to our result, previous studies showed that PCOM was present in 33%–54% of adolescents with regular menstrual cycles and this could be a feature of normal puberty [10, 11]. A study by Pena AS et al. reported that pelvic ultrasound cannot be considered ideal for the diagnosis of PCOS in those with a gynaecological age of <8 years (<8 years post menarche) due to the high incidence of multifollicular ovaries in this life stage [1]. In our study, we had defined polycystic ovaries as 12 or more follicles measuring 2–9 mm in diameter with or without an ovarian volume ≥10 mL, making group 3 as the most prevalent phenotype. Girls in this group had shown minimal deviation of metabolic and hormonal profiles compared to other groups in the study. These results in our study invite debate on the use of ultrasound as a criterion for defining PCOS in the adolescent population and recommended more comparative longitudinal community-based research for further conclusion.

As illustrated in Table 4, group 1, group 2, and group 3 hormonal values are compared. The independent variables such as serum LH, serum FSH, serum fasting glucose, serum fasting insulin, and serum testosterone are statistically significant among the groups. The post hoc test shows a significant difference between groups as described in Table 4. Similar to our results, the studies in [10, 11, 27] reported high LH, LH/FSH, S. testosterone, and BMI in MI + HA + PCOM and MI + HA cases compared to MI + PCOM or controls. They found no significant reference in FSH levels in different groups [10, 11, 27]. The hypothesis that explains our observations is that LH and insulin work together to increase serum androgen levels by stimulating androgen production from the ovary while lowering serum hormone-binding globulin levels [27]. Nonhyperandrogenic PCOS girls (group3) exhibited very little deviation in biochemical parameters emphasising that our observations support the literature that these individuals should be closely followed up until adulthood and revaluated if symptoms persist.

## 6. Conclusion

Abnormal menstruation in adolescents is a rising health problem. PCOS in adolescents is difficult not only in terms of the diagnosis but in treatment as well. While the adult criteria are used to define PCOS, there is usually a chance to over-diagnose of adolescent PCOS. Different endocrinopathies, which include thyroid dysfunction and hyperprolactinemia, have to be ruled out before establishing the diagnosis in adolescent girls. This analysis revealed that adolescent hyperandrogenism (phenotypes A and B) is associated with a more deranged hormonal and metabolic profile than nonandrogenic PCOS (phenotype D). To prevent long-term sequelae, lifestyle changes, early treatment, and close follow-up are recommended in this subset of girls.

In a nutshell, we state that further research needs to be carried out on a larger scale including women & girls belonging to different social, geographical, and economic statuses in order to clearly understand the hormonal profiles & ovarian morphologies among adolescent girls with menstrual irregularities which may aid in the diagnosis of adolescent PCOS.

### 6.1. Strength of the Study

This is one of the few Indian studies which aimed to evaluate the endocrine profile in adolescent girls with menstrual irregularities and comparatively analyzed different phenotypes of adolescent PCOS cases.

### 6.2. Limitations of the Study

This study was conducted in an urban tertiary care referral medical centre with the representing sample mostly belonging to upper socioeconomic status, either reported self or referred from specialty clinic, therefore the study cohort is less likely to be truly representative of the general population, thereby emphasising on the need of large-scale multicentric longitudinal studies to confirm our findings. Failure to demonstrate the comparability of the results due to the lack of a control group is another limitation observed in our study.

## Figures and Tables

**Table 1:** Demographic and clinical characteristics of study population.

S.N.	Demographic variables		*N* (%)	
1	Mean age^e^		17.08 ± 1.91	

2	Age at menarche^ea^		11.97 ± 1.025	

3	Socio economic status	Upper	9/248	03.62
		Upper-middle	80/248	32.25
		Lower-middle	99/248	39.91
		Upper-lower	43/248	17.33
		Lower	17/248	06.85

4	Family history of menstrual disorder	Present	86/248	34.67
		Absent	108/248	43.54
		Not sure	54/248	21.77

5	Menstrual complaint	Oligomenorrhoea	176/248	70.96
		Polymenorrhea	22/248	08.87
		Menorrhagia/menometrorrhagia	42/248	16.93
		Hypomenorrhoea	08/248	03.22

^a^data are shown as mean ± SD; ^b^data are shown as *n*(%).

**Table 2:** Endocrinal profile in adolescents with menstrual irregularities.

S. N.	Menstrual irregularities	Total no of cases (*n* = 248)	Thyroid dysfunction		Hyperprolactinemia		Biochemical hyperandrogenism	
			No (*n* = 21)	% (8.46%)	No (*n* = 3)	% (1.20%)	No (*n* = 37)	% (14.91%)
1.	Oligomenorrhoea	176	13	07.38	02	1.12	33	18.75
2.	Polymenorrhoea	22	02	09.09	0	0.0	01	04.54
3.	Menorrhagia	42	05	11.90	0	0.0	02	04.76
4.	Hypomenorrhoea	08	01	12.50	01	12.50	01	12.50

Data are shown as *n* (%)

**Table 3:** Clinical characteristics of different phenotypic groups of adolescents with menstrual irregularities.

Variables		Group- 1 *N* (%)	Group-2 *N* (%)	Group-3 *N* (%)	*p*-value
Family history of PCOS					
Present		26 (65.0)	15 (60.0)	20 (22.98)	0.05
Absent		14 (35.0)	10 (40.0)	67 (77.07)	

History of weight gain					
Present		20 (50.0)	14 (56.0)	16 (18.39)	0.001
Absent		20 (50.0)	11 (34.0)	71 (81.60)	

Hyperandrogenism					
Acne	Present	20 (50.0)	11 (44.0)	NA	
	Absent	20 (50.0)	14 (66.0)		
Hirsutism	Present	13 (32.5)	12 (48.0)		
	Absent	27 (67.5)	13 (52.0)		
Biochemical hyperandrogenemia	Present	16 (40.0)	09 (36.0)		
	Absent	24 (60.0)	16 (64.0)		

BMI ≥ 23					
Present		27 (67.5)	15 (60.0)	17 (19.54)	
Absent		13 (32.5)	10 (40.0)	70 (80.45)	

Ovarian morphology					
Bilateral PCOM		37 (92.5)	NA	67 (77.01)	
Right PCOM		03 (07.5)		14 (16.09)	
Left PCOM		0 (0.0)		6 (06.89)	

Group 1: MI + HA(C/B) + PCOM, group 2: MI + HA(C/B)-PCOM, and group:3 MI + PCOM -HA(C/B) data are shown as *n* (%); MI: menstrual irregularities; HA: hyperandrogenemia; C: clinical (moderate to severe acne/hirsutism mFG score ≥8,); B: biochemical (S. testosterone ≥82 ng/dl); PCOM: polycystic ovarian morphology, BMI: body mass index. *p* values were evaluated by using the chi-square test.

**Table 4:** Comparative analysis of endocrinal metabolic and hormonal parameters in different phenotypes of adolescent PCOS.

Variables	Group 1 mean ± SD (95% CI)	Group 2 mean ± SD (95% CI)	Group 3 mean ± SD (95% CI)	p-value	Bonferroni post hoc test (*p* < 0.05)
S.FSH mIU/ml	6.89 ± 1.85 (6.24–7.54)	7.09 ± 1.7 (5.96–8.23)	6.85 ± 2.72 (6.21`-7.49)	0.94	
S. LH mIU/ml	11.78 ± 2.56 (10.88–12.67)	13.10 ± 3.22 (11.05–15.15)	9.82 ± 3.25 (9.05–10.58	<0.001	1 vs 3, 2vs 3
BMI	23.91 ± 3.53 (22.78–25.04)	23.44 ± 3.64 (21.93–24.94)	20.53 ± 2.98 (19.90–21.17)	<0.001	1 vs 3, 2vs 3
S. fasting glucose mg/dl	89.72 ± 11.78 (85.73–93.70)	85.59 ± 11.31 (79.05–92.12)	80.05 ± 11.12 (77.52–82.57)	<0.001	1 vs 3
S. fasting insulin (*μ*IU/mL)	21.42 ± 4.26 (19.73–23.11)	23.58 ± 3.16 (21.45–25.71)	20.20 ± 3.93 (19.23–21.17)	0.026	2 vs 3
S. testosterone	77.24 ± 28.84 (67.18–87.30)	91.24 ± 30.75 (71.70–110.78)	47.22 ± 14.49 (43.81–50.62)	<0.001	1 vs3, 2 vs 3

Group 1: MI + HA(C/B) + PCOM, group 2: MI + HA(C/B)-PCOM, and group:3 MI + PCOM -HA(C/B) data are shown as mean ± SD; O: oligomenorrhoea; HA: hyperandrogenemia; C: clinical (moderate to severe acne/hirsutism mFG score ≥ 8; PCOM: polycystic ovarian morphology; S. FSH: serum follicular stimulating hormone; S. LH: Serum leutinizing hormone; BMI: body mass index. *p*-values were evaluated by one-way ANOVA; significance difference between any two group (*p* < 0.05), as demonstrated by the Bonferroni post hoc test.

## Data Availability

Data can be available from the corresponding author upon request.
